# On the synonymy of two *Acantholycosa* species (Araneae, Lycosidae) from the Altai

**DOI:** 10.3897/zookeys.559.7048

**Published:** 2016-02-03

**Authors:** Alexander A. Fomichev, Yuri M. Marusik, Seppo Koponen

**Affiliations:** 1 Altai State University, Lenina Pr., 61, Barnaul, RF-656049, Russia; 2 Institute for Biological Problems of the North RAS, Portovaya Str. 18, Magadan 685000, Russia; 3 Department of Zoology & Entomology, University of the Free State, Bloemfontein 9300, South Africa; 4 Far Eastern Federal University, Sukhanova 8, Vladivostok 690950, Russia; 5 Zoological Museum, University of Turku, FI-20014 Turku, Finland

**Keywords:** Aranei, wolf spider, South Siberia, Russia, new record, new synonymy

## Abstract

Two species previously known from East Kazakhstan, *Acantholycosa katunensis* Marusik, Azarkina & Koponen, 2004, known from the holotype male, and *Acantholycosa kurchumensis* Marusik, Azarkina & Koponen, 2004, **syn. n.** known from females, are synonymized, and priority is given to *Acantholycosa katunensis*. *Acantholycosa katunensis* is reported for the first time in the Russian Altai. Both sexes of this species are illustrated, and a distribution map is provided.

## Introduction

*Acantholycosa* Dahl, 1908 is one of the best studied among species-rich Pardosinae genera in the Holarctic due to a revision (Marusik et al. 2004), a regional review (Marusik and Omelko 2011) and several species surveys (Kronestedt and Marusik 2002; Fomichev and Marusik 2011; Marusik and Logunov 2011). Nevertheless, its taxonomy has remained improperly studied. Over one third of *Acantholycosa* species are known from only a single sex (two from females and eight from males). All these species are either endemic to the Altai-Sayan Mountain region (nine species) or the Sikhote-Alin’ Mountain Range (one species). Such a high number of species known only from one sex may be because of the habitat preference of *Acantholycosa* species. With one exception (*Acantholycosa lignaria* (Clerck, 1757)), all species inhabit stony screes in mountains, and this habitat is difficult to reach and study. So far, 29 species are known in the genus (World Spider Catalog 2015), most of which (20) occur in the Altai-Sayan Mountain region (Marusik et al. 2004).

While studying material from the Russian Altai, the first author found two species from nearby localities, *Acantholycosa katunensis* Marusik, Azarkina & Koponen, 2004 and *Acantholycosa kurchumensis* Marusik, Azarkina & Koponen, 2004, both previously known from East Kazakhstan. The former species, assigned to the *azyuzini*-group, was known from the holotype male from Rakhmanovskiye Klyuchi Village, and the latter species, assigned to the *dudkorum*-species group, was known from three females from two localities, the Kurchum River and Rakhmanovskiye Klyuchi. Marusik et al. (2004) suggested that these two species found in a single locality (Rakhmanovskiye Klyuchi) could be conspecific. The occurrence of these two species *Acantholycosa katunensis* and *Acantholycosa kurchumensis* in another locality led us to the conclusion that they are conspecific, and the tentative placement of these species into different species groups by Marusik et al. (2004) was incorrect. Additional arguments in support of the conspecificity of two species are the same spination of tibia I (5-6 proventral and 4-5 retroventral spines) and also that these species in South-Western Altai are the only known species by opposite sexes. The goal of this paper is to synonymize the two species, provide an illustrated redescription of both sexes and new data about species distribution.

## Material and methods

Specimens were photographed with a Canon EOS 7D camera attached to an Olympus SZX16 stereomicroscope at the Zoological Museum, University of Turku, Finland. Digital images were montaged using “CombineZP” image stacking software. Epigynes were cleared in a KOH/water solution until soft tissues were dissolved. Photographs were taken in dishes with paraffin on the bottom to hold the specimens in position. Background maps are from Microsoft Encarta Premium 2009. All material examined is deposited in the Institute for Systematic and Ecology of Animals, Novosibirsk (ISEA).

## Taxonomy

### Acantholycosa katunensis

Taxon classificationAnimaliaAraneaeLycosidae

Marusik, Azarkina & Koponen, 2004

Figs 1–2 , 3–10

Acantholycosa katunensis Marusik, Azarkina & Koponen, 2004: 107, figs 21–23 (♂).
Acantholycosa kurchumensis Marusik, Azarkina & Koponen, 2004: 119, figs 82–83 (♀). **Syn. n.**

#### Material examined.

RUSSIA, ***Altai*** Republic, Kosh-Agach District: 3♂ (ISEA), Karagemskyi Mt. Range (49°52’N; 87°07’E), 2500–2900 m, “kurums” (=scree formed by huge boulders) and alpine meadow, 27.06.2014 (A.A. Fomichev); 1♀ (ISEA), Karagem River valley (49°53’N; 87°11’E ), 1360 m, stony steppe slope, 28.06.2014 (A.A. Fomichev); KAZAKHSTAN, ***East Kazakhstan*** Area: 1♂ (holotype of *Acantholycosa katunensis*) (ISEA) South Altai, south part of Katun’ Mt. Range, 5 km SE of Rakhmanovskiye Klyuchi (=Springs), 2100–2500 m, alpine zone, 26.06.1997 (R.Yu. Dudko and V.K. Zinchenko); 1♀ (holotype of *Acantholycosa kurchumensis*) (ISEA) Kurchum Mt. Range, Kurchum River, upper flow, 23.08.1990 (V.K. Zinchenko).

#### Diagnosis.

*Acantholycosa katunensis* is most similar to *Acantholycosa dudkorum* Marusik, Azarkina & Koponen, 2004 by having a similarly shaped tegular apophysis that lacks an apical arm, a similar conductor, a wide apical pocket and a thin septum. The two species can be separated by the shape of the embolus, which tapers toward the tip in *Acantholycosa katunensis* (Figs 3–5) and widens in *Acantholycosa dudkorum* (cf. Marusik et al. 2004: figs 73, 75); the shape of the palea: in *Acantholycosa dudkorum* (cf. Marusik et al. 2004: figs 73, 75), the paleal process is thin with a hollow on the prolateral side, and it is unmodified in *Acantholycosa katunensis* (Figs 4–5, 9). Females of the two species can be distinguished by the shape of septum: long (starting from the pocket) with a subparallel base in *Acantholycosa katunensis* and short (starting from fovea) and widened at the base in *Acantholycosa dudkorum* (cf. Marusik et al. 2004: fig. 78).

**Figures 1–2.**
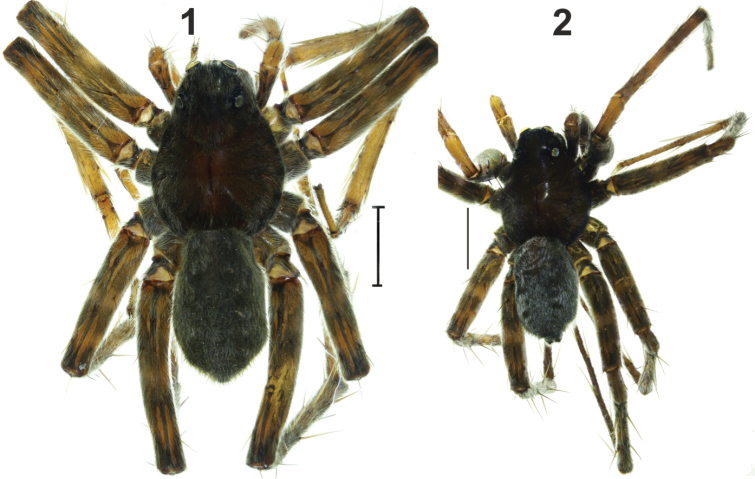
Habitus of *Acantholycosa katunensis*. **1** female **2** male. Scale: 2 mm.

**Figures 3–10.**
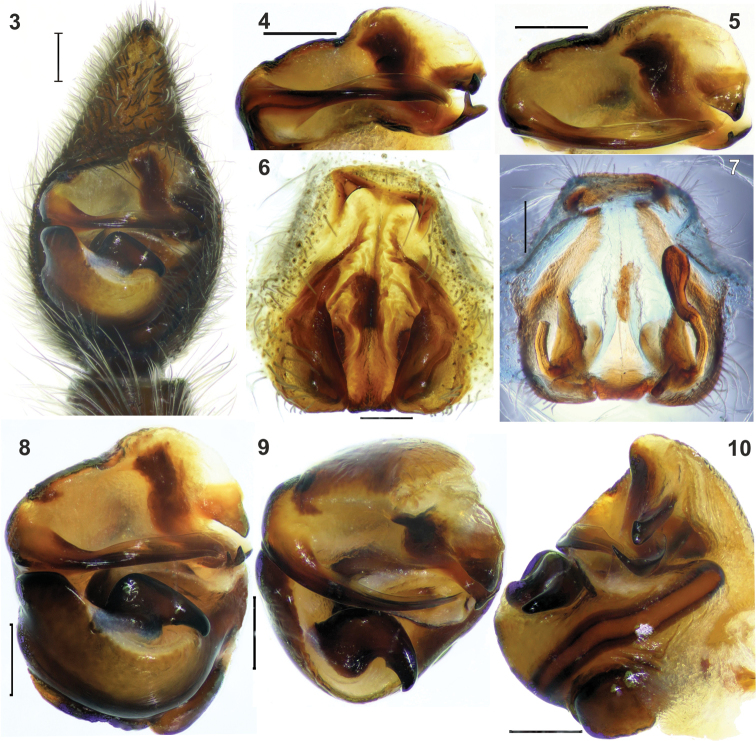
Copulatory organs of *Acantholycosa katunensis*. **3** male palp, ventral **4–5** embolic division, ventral and anterior **6–7** epigyne, ventral and dorsal (left head of receptacle is broken) **8–10** bulb, ventral, anterior and retrolateral. Scale: 0.2 mm.

#### Description.

See Marusik et al. (2004).

#### Distribution.

**Map 1.**
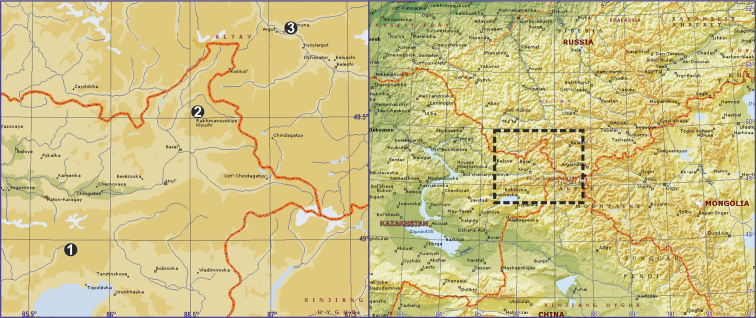
Distribution records of *Acantholycosa katunensis*. **1** Kurchum River **2** Rakhmanovskiye Klyuchi Village **3** Karagemskyi Mt. Range.

So far, this species is known from three localities. The most distant localities, the upper reaches of the Kurchum River (locality 1, Map 1) and the Karagemskyi Mt Range (locality 3) are approximately 190 km apart (Map 1). Rakhmanovskiye Klyuchi Village (locality 2), the type locality of *Acantholycosa katunensis*, is located between two extreme distribution records (Map 1). Listings for the species by Platnick (2004–2014) and the World Spider Catalog (2015) for Russia were erroneous based on wrong country data provided by Marusik et al. (2004). Type locality of *Acantholycosa katunensis* was mistakenly assigned to Russia (Altai) by Marusik et al (2004) instead of Kazakhstan (East Kazakhstan Area). The new record from the Karagemskyi Mt Range is the first locality record of this species in Russia.

## Discussion

Synonymizing these two names reduced the number of species known in the genus to 27. Although the number of *Acantholycosa* species in the Altai-Sayan Mountain Region decreased from 20 to 19, this region still has the highest species diversity and endemism (59%) in the entire range of the genus. The record of *Acantholycosa katunensis* from the Russian Altai increased the number of *Acantholycosa* species found in Russia from 21 to 22. Judging from the high level of endemism in the Altai-Sayan Mountain Region, the limited distribution of species in that area, habitat preferences (stony screes) and the number of unexplored mountain ranges in the West and East Sayan Mountains (cf. Marusik et al. 2004: map 1) and the lack of material from adjacent Xinjiang (China) and Mongolia, many new species are expected.

## Supplementary Material

XML Treatment for Acantholycosa katunensis
